# Severe Nonhepatic Hyperammonemia Associated With Ureaplasma parvum and Mycoplasma hominis Infection in a Dually Immunosuppressed Woman Presenting As Suspected Peritoneal Carcinomatosis

**DOI:** 10.7759/cureus.114045

**Published:** 2026-08-06

**Authors:** Ary De Sousa, Ana Cysneiros

**Affiliations:** 1 Neurocritical Care Unit, Unidade Local de Saúde de São José, Lisbon, PRT; 2 Medical Emergency Unit, Unidade Local de Saúde de São José, Lisbon, PRT

**Keywords:** biologic therapy, continuous renal replacement therapy, immunosuppression, molecular diagnosis, mycoplasma hominis, nonhepatic hyperammonemia, polyserositis, ureaplasma parvum

## Abstract

Severe nonhepatic hyperammonemia is a rare but potentially life-threatening condition in immunocompromised patients. *Ureaplasma* and *Mycoplasma* species are fastidious urogenital organisms that generate ammonia and may cause disseminated, culture-negative infection. We report the case of a 63-year-old woman receiving dual biologic therapy, ocrelizumab for multiple sclerosis and secukinumab for psoriasis, who presented with a history of sterile pyuria and lower urinary tract symptoms. Within two months, her condition progressed to polyserositis, paralytic ileus, multiorgan dysfunction, and metabolic encephalopathy requiring admission to intensive care. Imaging suggested a left ovarian neoplasm with peritoneal carcinomatosis, prompting exploratory laparotomy, which ultimately revealed diffuse seropurulent pachyperitonitis and a small localized purulent collection, without evidence of malignancy. Plasma ammonia rose despite the absence of hepatic failure, causing severe encephalopathy requiring continuous renal replacement therapy (CRRT). Conventional cultures of urine, blood, ascitic fluid, vaginal exudate, and peritoneal specimens were repeatedly negative, prompting investigation for unusual organisms. Urine polymerase chain reaction ultimately detected *Ureaplasma parvum* and *Mycoplasma hominis*. Plasma ammonia remained dependent on CRRT, with recurrent rebound following interruptions before targeted antimicrobial therapy was established. Following treatment with levofloxacin and doxycycline, sustained metabolic control allowed discontinuation of CRRT without further rebound hyperammonemia. The temporal pattern, while occurring alongside surgical drainage and peritoneal lavage, supports targeted treatment of the presumed underlying invasive infection as an important component of recovery. This case highlights the importance of considering fastidious urogenital organisms in immunosuppressed patients with unexplained nonhepatic hyperammonemia and persistently negative conventional cultures, particularly when ammonia levels remain dependent on extracorporeal clearance.

## Introduction

Hyperammonemia is most often a consequence of hepatic failure. However, it may also arise in nonhepatic contexts, where it is frequently underrecognized and carries a high risk of cerebral edema and death if not promptly addressed [1,2]. In adults, nonhepatic hyperammonemia may result from impaired ammonia clearance, including late-onset urea-cycle disorders, particularly partial ornithine transcarbamylase deficiency, and portosystemic shunting, or from increased ammonia production associated with urease-producing urinary tract infections, particularly in the setting of urinary stasis or obstruction, drug-related causes, severe catabolic states, and an increased intestinal nitrogen load, such as gastrointestinal bleeding [3].

Among infectious causes, urease-producing or arginine-metabolizing organisms are increasingly described in immunocompromised hosts [4]. *Ureaplasma* species and *Mycoplasma hominis* are urogenital commensals that lack a cell wall and are not reliably recovered by routine bacterial cultures. *Ureaplasma* hydrolyzes urea through urease activity, whereas *Mycoplasma hominis* derives energy from arginine, and both pathways generate ammonia [4-6]. Disseminated infection by these organisms has been reported as a cause of fatal hyperammonemia, most notably in lung transplant recipients and in patients treated for hematologic malignancies [1,4,5].

In lung transplant recipients, hyperammonemia syndrome has been reported in approximately 1-4% of patients. Mortality was 57.5% in a systematic review, while earlier series reported fatality rates exceeding 75% [7]. Across two donor-screening cohorts, *Ureaplasma* species were detected in approximately 10.8% of donor lungs [8]. Although the reported experience is concentrated among lung transplant recipients, the syndrome has also been described in other immunocompromised populations, particularly patients with hematologic malignancies receiving intensive immunosuppressive therapies [8,9]. Because these fastidious organisms are not reliably detected by routine bacterial culture, diagnosis generally requires targeted culture or nucleic acid amplification testing [10].

We describe a patient receiving dual biologic immunosuppression who developed severe nonhepatic hyperammonemia, polyserositis, and an imaging appearance mimicking peritoneal carcinomatosis, in whom urine polymerase chain reaction (PCR) detected *Ureaplasma parvum* and *Mycoplasma hominis*. This case emphasizes both the diagnostic difficulty and the therapeutic implications of this syndrome.

## Case presentation

A 63-year-old woman, previously independent, presented with a two-month history of urinary urgency, frequency, occasional hematuria, and hypogastric pain. Her medical history included relapsing multiple sclerosis treated with ocrelizumab (anti-CD20) since 2022, with the most recent infusion administered three months before hospital admission, and psoriasis treated with secukinumab (anti-interleukin-17A), with the most recent dose administered four weeks before admission. She also had a history of nephrolithiasis, and a previous ultrasound had demonstrated a non-obstructing 9-mm calculus in the left kidney. Over the preceding weeks, she had received several empirical antibiotic courses, including fosfomycin, nitrofurantoin, cefuroxime, and co-trimoxazole, at different emergency departments without clinical improvement. Urine cultures remained repeatedly negative despite persistent pyuria.

She was admitted with suspected left pyelonephritis and started on empirical piperacillin/tazobactam. Fever subsequently recurred, and computed tomography demonstrated left ureterohydronephrosis without an obstructing calculus. She underwent cystoscopy and left ureteral stent placement; diffuse erythema of the bladder wall was noted intraoperatively, and renal pelvic urine was sterile. Her condition did not improve, and she developed bilious vomiting, food intolerance, and rising inflammatory markers. Thoraco-abdomino-pelvic computed tomography demonstrated increasing ascites and bilateral adnexal masses with features suspicious for malignancy.

She subsequently developed progressive polyserositis, comprising ascites, bilateral pleural effusions, and a thin pericardial effusion, together with peritonitis of unknown cause, paralytic ileus, and progressive acute kidney injury. Broad-spectrum therapy was escalated to meropenem, and fluconazole was added when presumptive fungal cystitis was considered. Her level of consciousness progressively deteriorated, and the first measured plasma ammonia concentration was already elevated at 199 µmol/L. Despite treatment with lactulose, rifaximin, prokinetic agents, and nasogastric decompression, both the hyperammonemia and encephalopathy worsened. She was subsequently admitted to the intensive care unit (ICU) with type 1 respiratory failure and progressive metabolic encephalopathy.

On ICU admission, she was encephalopathic with a Glasgow Coma Scale score of 9 and West Haven grade III-IV, and plasma ammonia had increased to 575 µmol/L. She was tachypneic at 25-30 breaths/min, with bibasilar crackles on auscultation, and respiratory support with a high-flow nasal cannula was initiated. Abdominal examination revealed distension and tenderness. Laboratory studies at ICU admission showed leukocytes of 13.3 × 10⁹/L, including neutrophils of 11.3 × 10⁹/L, and a C-reactive protein concentration of 347.7 mg/L. Although these values had decreased from preceding ward peaks of 40.3 × 10⁹/L and 449.9 mg/L, respectively, following prolonged broad-spectrum antimicrobial therapy, this partial biochemical improvement did not parallel her clinical course, which continued to deteriorate. There was no evidence of hepatic failure sufficient to explain the hyperammonemia. Cross-sectional imaging showed a patent portal venous system without evidence of portosystemic shunting. Selected laboratory results are summarized in Table 1. Illness severity at ICU admission was substantial, with a Simplified Acute Physiology Score II of 37, corresponding to a predicted mortality of 19.6%, and an Acute Physiology and Chronic Health Evaluation II score of 13.

**Table 1:** Selected laboratory parameters at ward admission, during pre-ICU deterioration, and at ICU admission * Selected pre-ICU values represent the most clinically relevant peak or nadir documented during deterioration on the ward and were not necessarily obtained from the same blood sample. ‡ Plasma ammonia was not measured at ward admission; the first determination obtained before ICU transfer was already elevated at 199 µmol/L. ALP: alkaline phosphatase, ALT: alanine aminotransferase, AST: aspartate aminotransferase, eGFR: estimated glomerular filtration rate, GGT: gamma-glutamyl transferase, ICU: intensive care unit, INR: international normalized ratio

Parameter	Ward admission	Selected pre-ICU value*	ICU admission	Reference range
Leukocytes (×10⁹/L)	13.2	40.3	13.34	4.5-11.0
Neutrophils (×10⁹/L)	11.8	37.9	11.30	2.0-8.5
Hemoglobin (g/dL)	10.0	7.8	7.5	12.0-15.0
Platelets (×10⁹/L)	274	380	417	150-450
C-reactive protein (mg/L)	342	449.9	347.7	<5
Procalcitonin (ng/mL)	0.42	5.22	0.55	<0.05
Creatinine (mg/dL)	1.46	2.56	1.67	0.52-0.95
eGFR (mL/min/1.73 m²)	38	19	32	>60
Albumin (g/L)	24.7	21.7	17.2	32-46
ALT (U/L)	51	37	32	<33
AST (U/L)	105	94	94	<32
GGT (U/L)	-	179	260	<40
ALP (U/L)	81	128	123	35-104
Total bilirubin (mg/dL)	0.18	0.46	0.19	<1.2
INR	1.18	1.24	0.97	0.8-1.2
Plasma ammonia (µmol/L)	-	199‡	575	16-60

**Figure 1:**
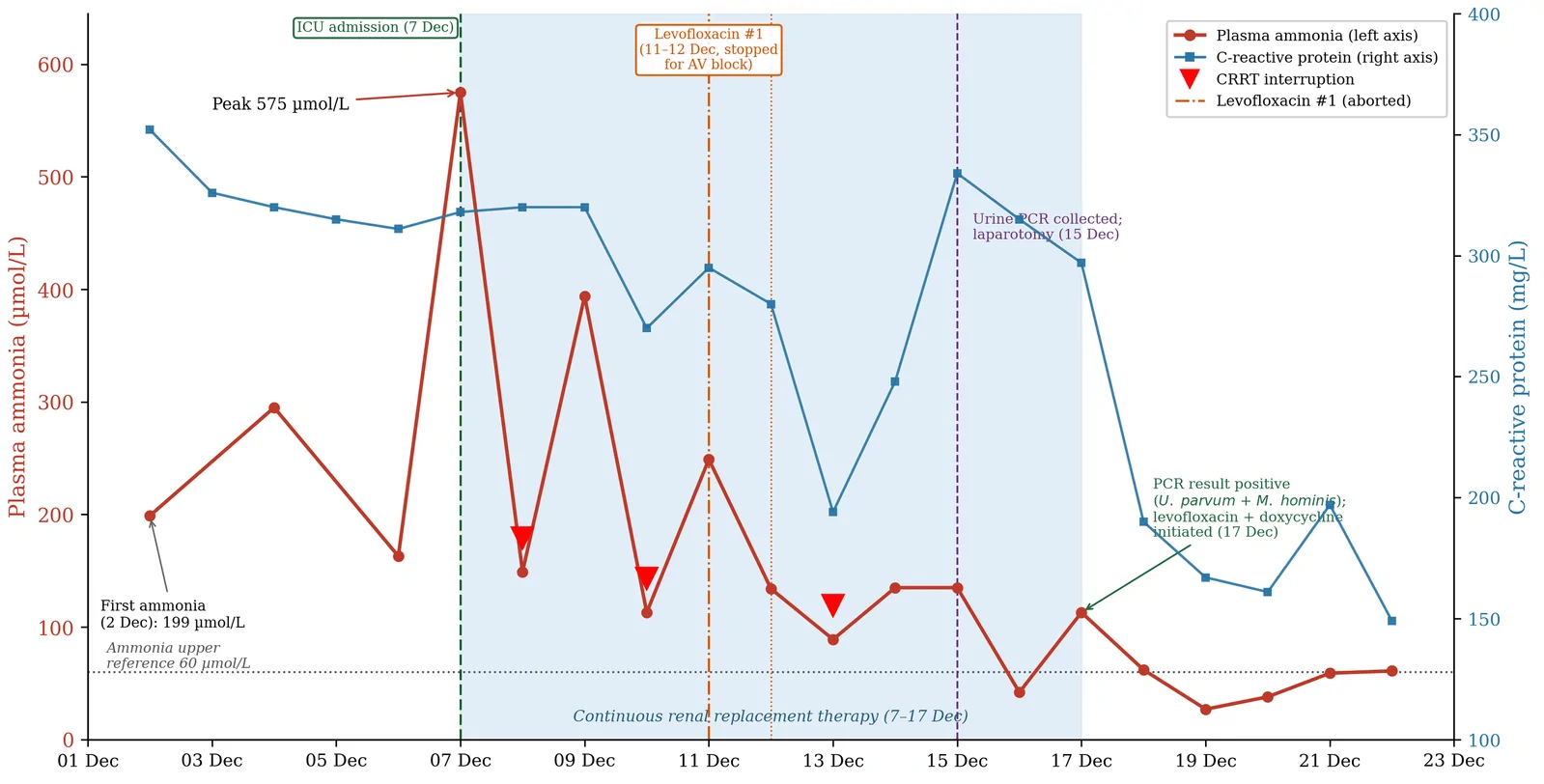
Plasma ammonia and C-reactive protein trajectories during the hyperammonemic phase of hospitalization Plasma ammonia concentrations are shown in red on the left axis and C-reactive protein levels in blue on the right axis. The plotted interval begins with the first plasma ammonia measurement during pre-ICU deterioration and does not encompass the earlier ward-admission period represented in Table 1; accordingly, the first C-reactive protein value shown in the figure is not the ward-admission value. Plasma ammonia was first measured before ICU admission at 199 µmol/L and peaked at 575 µmol/L around the time of transfer. The shaded area represents CRRT from 7 to 17 December. Red inverted triangles indicate documented CRRT interruptions followed by ammonia rebounds to 394, 249, and 135 µmol/L. A brief empirical course of levofloxacin on 11-12 December was discontinued as a precaution during episodes of high-grade atrioventricular block. A urine sample for PCR testing was collected, and an exploratory laparotomy was performed on 15 December. The positive result for *Ureaplasma parvum* and *Mycoplasma hominis* became available on 17 December, when targeted therapy with levofloxacin and doxycycline was initiated and CRRT was discontinued. No further rebound occurred, and plasma ammonia subsequently normalized within the laboratory reference range of 16-60 µmol/L. The dotted line marks the upper reference limit for plasma ammonia. AV: atrioventricular, CRRT: continuous renal replacement therapy, ICU: intensive care unit, PCR: polymerase chain reaction

Given the rapid increase in plasma ammonia and worsening level of consciousness, continuous renal replacement therapy (CRRT) was initiated for extracorporeal ammonia clearance. Continuous veno-venous hemodiafiltration was delivered using regional citrate anticoagulation, an Ultraflux AV1000S hemofilter on a Fresenius multiFiltrate PRO platform, a blood-flow rate of 80 mL/min, and a prescribed effluent dose of 25-50 mL/kg/h. During the initial phase, ammonia control remained dependent on extracorporeal clearance: CRRT was interrupted on several documented occasions, each followed by an abrupt rebound in plasma ammonia. The complete ammonia trajectory in relation to CRRT and the other key interventions is shown in Figure 1.

Differential diagnosis and investigations

The differential diagnosis of polyserositis in this immunosuppressed patient included bacterial, fungal, mycobacterial, and viral infection, autoimmune serositis, and malignancy. In parallel, the differential diagnosis of nonhepatic hyperammonemia included a late-onset inborn error of metabolism, medication-related causes, and increased nitrogen load from occult gastrointestinal bleeding. No exposure to drugs known to cause hyperammonemia was identified. Over the 14 days between ward and ICU admission, hemoglobin decreased from 10.0 to 7.5 g/dL. However, there was no clinical evidence of overt gastrointestinal bleeding, and computed tomography angiography showed no intra-abdominal hemorrhage. In the context of prolonged critical illness, the decrease in hemoglobin was considered more consistent with multifactorial anemia than with a clinically significant gastrointestinal nitrogen load; gastrointestinal bleeding was therefore considered an unlikely major contributor to the hyperammonemia.

Given her exposure to dual biologic therapy, immunological assessment was performed. Lymphocyte subset analysis demonstrated profound and selective B-cell depletion, with CD19+ B-cells of 1.29 cells/µL (0.10%; reference range, 90-660 cells/µL), while total T-cell and natural killer-cell counts remained within their respective reference ranges, consistent with the expected pharmacodynamic effect of anti-CD20 therapy.

An autoimmune panel was negative, including antinuclear antibodies, extractable nuclear antigen antibodies, anti-double-stranded DNA antibodies, antineutrophil cytoplasmic antibodies, and anti-glomerular basement membrane antibodies. Serum IgG4 was within the reference range.

Tumor-marker assessment showed an elevated CA-125 level of 177 U/mL and a mildly elevated CA-15-3 level of 30 U/mL, reinforcing the initial concern for ovarian malignancy.

A late-onset inborn error of metabolism was actively investigated during the hyperammonemic crisis. Plasma amino acid analysis obtained during active hyperammonemia showed no pattern suggestive of a primary urea-cycle disorder: glutamine, citrulline, and arginine were within their respective reference ranges, while ornithine was marginally reduced. Urinary orotic acid was mildly elevated at 5.33 µmol/mmol creatinine (reference range, 0.1-1.9 µmol/mmol creatinine). Total and free carnitine concentrations were mildly reduced, 33 µmol/L (reference range, 37-92 µmol/L) and 22 µmol/L (reference range, 26-90 µmol/L), respectively, with a preserved free-to-total carnitine ratio of 67% (reference, >50%). These findings were considered nonspecific in the context of critical illness and parenteral nutrition. Urinary organic acid chromatography showed no specific abnormality. Genetic testing was not pursued because the biochemical profile obtained during active hyperammonemia did not support a primary urea-cycle disorder, and the clinical context, including a presumed infectious trigger, absence of hepatic failure sufficient to explain the hyperammonemia, no previous hyperammonemic episodes, and complete resolution without recurrence, also argued against this diagnosis.

**Figure 2:**
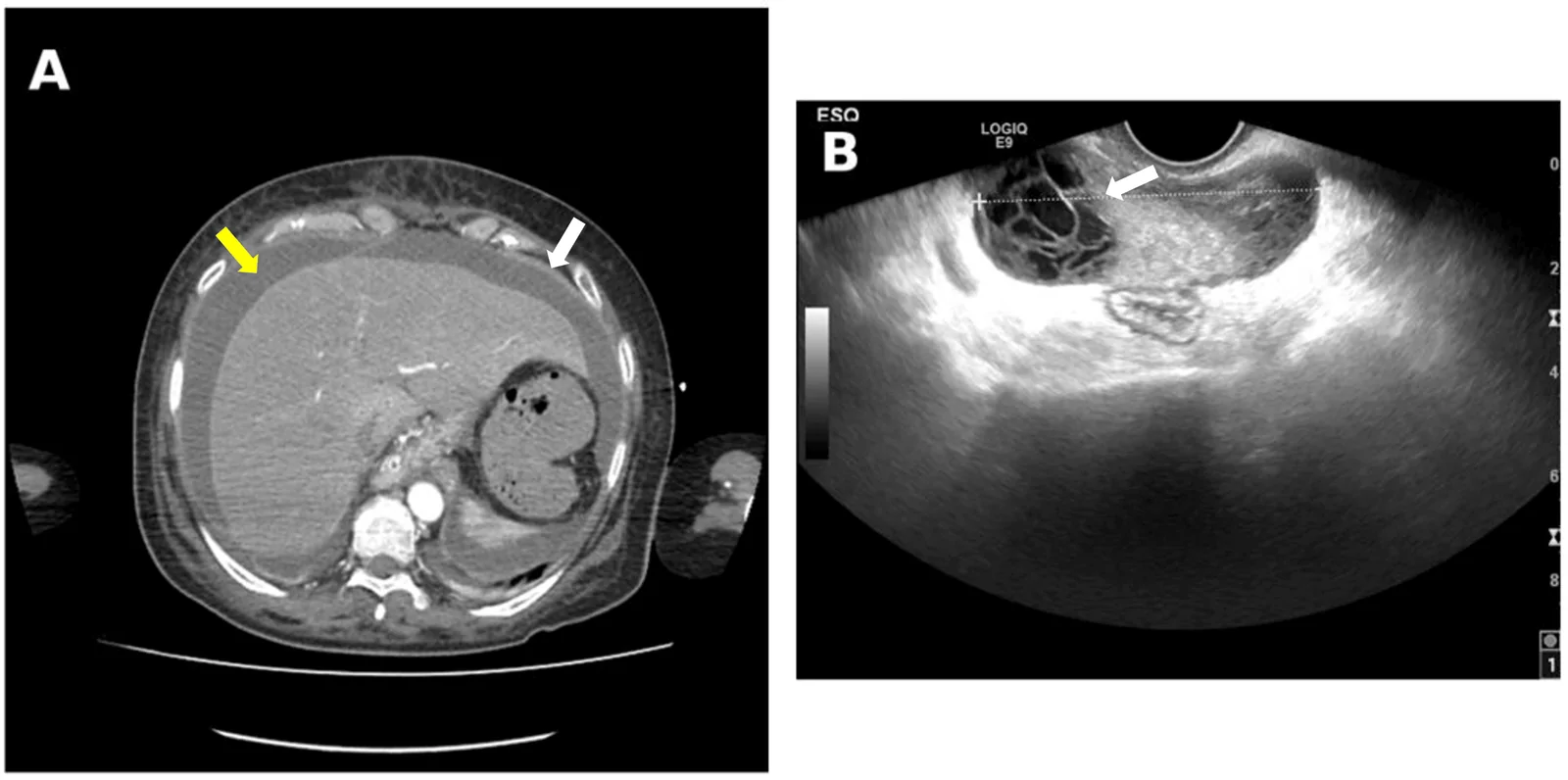
Imaging findings initially suggesting malignancy (A) Axial contrast-enhanced computed tomography showing voluminous ascites (yellow arrow) and diffuse peritoneal thickening with enhancement (white arrow), initially raising concern for peritoneal carcinomatosis. (B) Pelvic ultrasound demonstrating a multiseptated, multicystic left ovary (white arrow), interpreted as a possible ovarian neoplasm.

**Figure 3:**
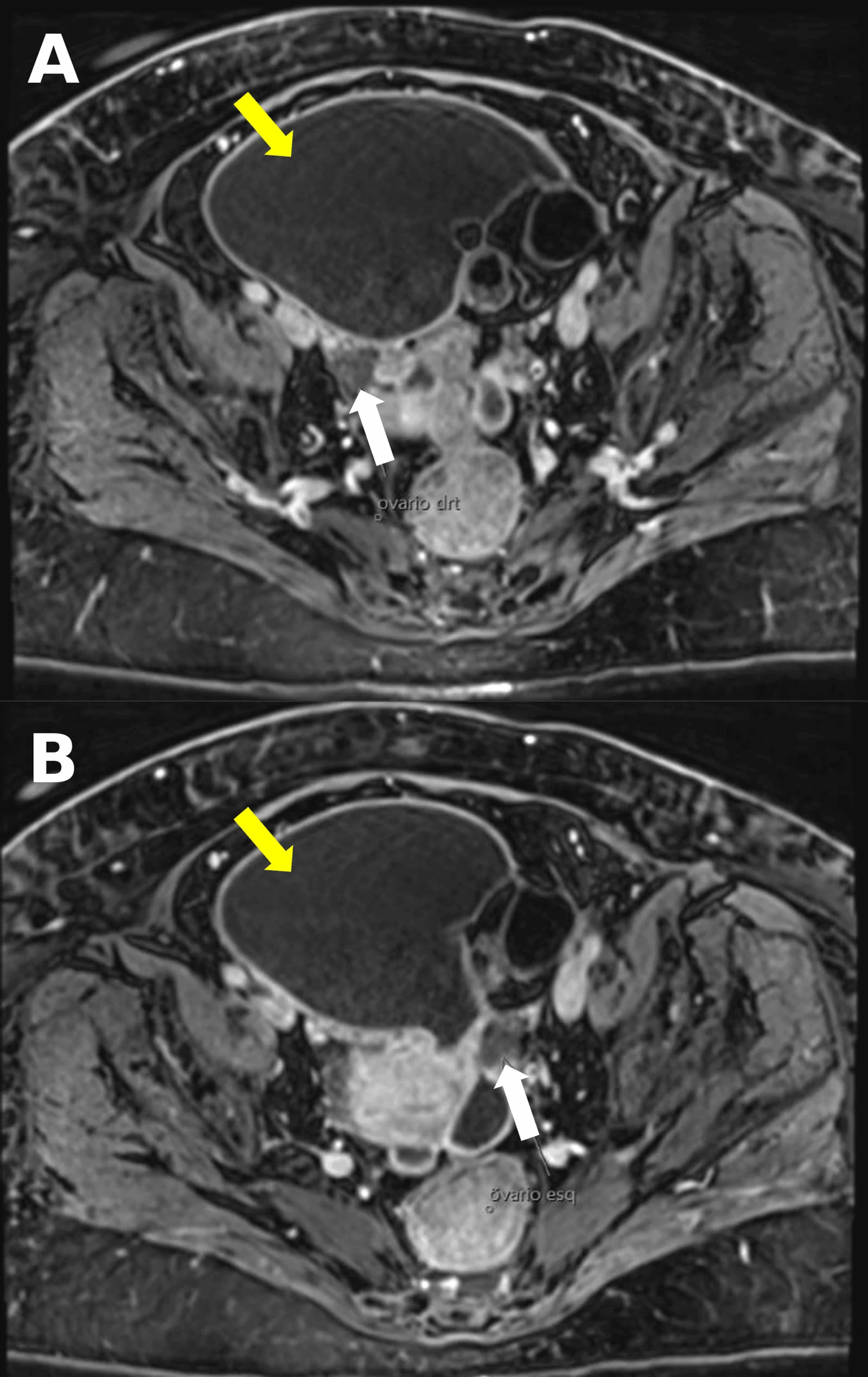
Contrast-enhanced fat-suppressed pelvic magnetic resonance imaging demonstrating morphologically normal ovaries (A) Morphologically normal right ovary (white arrow), with ascites (yellow arrow). (B) Morphologically normal left ovary (white arrow), with ascites (yellow arrow). The normal ovarian morphology, together with the operative, cytological, and histological findings, strongly argued against ovarian or peritoneal malignancy.

Sequential computed tomography and ultrasonography demonstrated progressive ascites, peritoneal thickening, pleuropericardial effusions, left pelvicalyceal dilatation, and a 65-mm left adnexal lesion with solid and multiseptated cystic components. This imaging appearance was initially interpreted as a probable ovarian neoplasm with peritoneal carcinomatosis (Figure 2). Pelvic magnetic resonance imaging subsequently demonstrated loculated ascites with peritoneal thickening and enhancement consistent with peritonitis, diffuse bladder-wall thickening, and morphologically normal ovaries, strongly arguing against ovarian malignancy (Figure 3). Cranial magnetic resonance imaging showed only the previously known demyelinating lesions, without mass effect or abnormal enhancement.

Repeated conventional cultures of urine, blood, ascitic fluid, vaginal exudate, and peritoneal specimens remained negative. Ascitic fluid analysis showed a polymorphonuclear-predominant exudate with an elevated adenosine deaminase level of 68.6 U/L. Cytological examination was performed both before surgery, using fluid obtained by ultrasound-guided paracentesis, and intraoperatively, using additional ascitic fluid and fibrin specimens; both evaluations were negative for malignant cells.

A comprehensive investigation for tuberculosis was unrevealing. Repeated acid-fast bacilli smears of ascitic fluid were negative, and mycobacterial cultures of both ascitic fluid and peritoneal tissue yielded no growth. Mycobacterial blood cultures also remained negative after 42 days of incubation. Nucleic acid amplification testing for *Mycobacterium tuberculosis* complex was negative in both ascitic fluid and urine, and the interferon-gamma release assay was negative. Peritoneal and appendiceal histological examination showed no granulomas or caseating necrosis. Taken together, these findings strongly argued against tuberculous peritonitis.

Given the persistent sterile pyuria, repeatedly negative conventional cultures, and severe hyperammonemia, targeted urine PCR testing for *Ureaplasma parvum*, *Ureaplasma urealyticum*, and *Mycoplasma hominis* was requested. The result became available on 17 December and detected *Ureaplasma parvum* and *Mycoplasma hominis*.

Exploratory laparotomy and pathology

Because of the persistent inflammatory response and concern for peritoneal carcinomatosis, exploratory laparotomy was performed. Operative findings included diffuse seropurulent peritonitis with fibrinous plaques at different stages of organization and extensive adhesions, producing a pachyperitonitis-like appearance throughout the abdominal cavity. A small localized collection containing frank purulent material was identified adjacent to the hepatic flexure of the colon, without evidence of gastrointestinal perforation. The collection was drained, followed by peritoneal lavage. Extensive adhesiolysis was technically demanding and required multiple enterorrhaphies and repair of the rectal serosa. Conventional bacterial culture of the purulent material drained from the collection yielded no growth.

Histological examination of the ileocecal appendix showed fibroadipose involution with a fibrinogranulocytic serosal exudate, consistent with an extrinsic inflammatory process; a second fragment was necrotic and non-diagnostic. No granulomas or caseating necrosis were identified. Ascitic fluid cytology showed a fibrinous background containing abundant inflammatory cells and rare reactive mesothelial cells, without malignant cells. Together with the morphologically normal ovaries on magnetic resonance imaging and the absence of malignancy on cytological and histological examination, these findings strongly argued against an underlying ovarian or peritoneal malignancy.

Treatment

Antimicrobial therapy initially comprised piperacillin/tazobactam, followed by meropenem and fluconazole, all without microbiological yield. When infection with an ammonia-producing microorganism was first suspected, empirical levofloxacin was initiated. However, it was discontinued after 24 hours as a precaution because of recurrent episodes of high-grade atrioventricular block with prolonged pauses. These episodes had begun during dexmedetomidine infusion and had already prompted withdrawal of that agent. After urine PCR detected *Ureaplasma parvum* and *Mycoplasma hominis*, targeted therapy with levofloxacin (750 mg every 24 hours) and doxycycline (100 mg every 12 hours) was initiated and continued for 14 days. Treatment was completed without recurrence of the conduction disturbance or other complications. A concurrent nosocomial tracheobronchitis caused by *Stenotrophomonas maltophilia* was treated separately.

Outcome and follow-up

Ammonia clearance initially remained dependent on CRRT. Sustained metabolic control and neurological recovery followed surgical drainage and peritoneal lavage, molecular identification of the presumed pathogens, and initiation of targeted antimicrobial therapy, allowing CRRT to be discontinued without further rebound.

Renal function normalized, and respiratory support was progressively weaned to low-flow nasal oxygen. Follow-up contrast-enhanced computed tomography showed only minimal residual peritoneal fluid with mild enhancement of the peritoneal leaflets, representing a marked improvement from the initial studies. Urine PCR performed after completion of antimicrobial therapy was negative for both *Ureaplasma* and *Mycoplasma*. The patient was discharged for intensive rehabilitation. At approximately eight months of follow-up, she was ambulatory with a cane and independent in activities of daily living, despite mild residual distal weakness of the left lower limb.

## Discussion

This case illustrates the diagnostic and therapeutic challenges of nonhepatic hyperammonemia associated with fastidious urogenital organisms in a profoundly immunosuppressed patient. Two features make it particularly instructive. First, the patient was receiving two biologic agents with distinct mechanisms: ocrelizumab, which depletes CD20-positive B-cells, and secukinumab, which blocks interleukin-17A. The degree of B-cell depletion was documented and profound, with a CD19+ B-cell count of 1.29 cells/µL, approximately 1% of the lower reference limit. At the same time, T-cell and natural killer-cell compartments were preserved. Anti-CD20 therapy can lead to secondary hypogammaglobulinemia and is associated with an increased risk of serious and atypical infections in patients with multiple sclerosis [11]. Interleukin-17A blockade modifies mucosal antimicrobial defenses, and consensus guidance identifies increased susceptibility to selected infections as a relevant consideration for this class of biologic agents [12]. The combined use of two agents acting on distinct immune pathways may therefore have created a plausible substrate for invasive disease caused by organisms that are ordinarily low-grade urogenital commensals. However, the individual contribution of each agent cannot be determined. Second, the clinical and imaging findings initially pointed strongly toward peritoneal carcinomatosis, prompting surgical exploration before the presumed infectious etiology was established.

Beyond solid-organ transplantation and hematologic malignancy, invasive *Ureaplasma* and *Mycoplasma* infections are increasingly reported in patients receiving B-cell-depleting therapies and in those with other forms of humoral immunodeficiency. Invasive *Ureaplasma* disease has been described in rituximab recipients [13], as well as in an individual with multiple sclerosis treated with ocrelizumab [14]. Fatal nonhepatic hyperammonemia has likewise been reported following treatment with the CD20-directed bispecific antibody glofitamab [15]. These observations place the present case within an emerging pattern in which profound B-cell dysfunction associated with anti-CD20 and related therapies may predispose to invasive disease caused by ammonia-generating urogenital organisms.

The combination of persistent sterile pyuria, repeatedly negative conventional cultures, and severe hyperammonemia in the absence of hepatic failure sufficient to explain it provided the decisive diagnostic clue. In this setting, urease-producing and arginine-metabolizing organisms should be considered early because routine bacterial cultures do not reliably detect them, and targeted culture or nucleic acid amplification testing is generally required [10]. *Ureaplasma* species and *Mycoplasma hominis* have been implicated in hyperammonemia syndrome in immunocompromised hosts, classically after lung transplantation and during treatment for hematologic malignancies [1,4,5,7]. The present case reinforces that the presumed source need not be pulmonary and may instead be urogenital or abdominopelvic [8].

The ability of this condition to mimic malignancy represents another important diagnostic lesson. The combination of elevated CA-125 and ascites is not specific for ovarian cancer. Comparable presentations with marked ascites, adnexal abnormalities, peritoneal thickening, and CA-125 elevation have been described in benign inflammatory, infectious, and autoimmune serosal disorders and may be radiologically indistinguishable from peritoneal carcinomatosis [16]. In the present case, the elevated ascitic-fluid adenosine deaminase level also raised concern for tuberculous peritonitis. However, the extensive negative mycobacterial investigation and the absence of granulomas or caseating necrosis on histological examination strongly argued against this diagnosis. This recognized diagnostic pitfall supports pursuing a comprehensive infectious and inflammatory work-up before attributing polyserositis and peritoneal abnormalities to a neoplastic process.

Management hinges on two parallel priorities: rapid reduction of plasma ammonia and treatment of the presumed underlying infection. Nonhepatic hyperammonemia may progress to seizures, cerebral edema, coma, and death within hours, and status epilepticus and fatal cerebral edema have been reported in disseminated *Ureaplasma* and *Mycoplasma* infections [17]; it is, nonetheless, potentially reversible with prompt recognition and treatment [18]. Extracorporeal removal is therefore central to acute control in severe cases with rapidly increasing ammonia concentrations or neurological deterioration despite conservative measures. In the present patient, metabolic control initially remained dependent on CRRT, with documented interruptions followed by abrupt ammonia rebounds. Concurrent acute kidney injury may also have impaired renal ammonia elimination and amplified the severity of the syndrome, providing further rationale for early extracorporeal support [19]. Consensus recommendations endorse extracorporeal ammonia clearance in severe hyperammonemia refractory to conservative treatment [20].

Surgical drainage of the localized purulent collection and peritoneal lavage may have contributed to source control. Once the urine PCR result became available, targeted antimicrobial therapy with levofloxacin and doxycycline was initiated, CRRT was discontinued without further rebound, and plasma ammonia subsequently normalized. Because *Ureaplasma* and *Mycoplasma hominis* lack a cell wall, beta-lactam antibiotics are ineffective, whereas tetracyclines and fluoroquinolones are commonly used. Susceptibility may vary, however, and antimicrobial susceptibility testing was not available in this case. The 14-day course was selected in accordance with durations commonly reported for invasive infection rather than a validated treatment protocol.

The principal limitation is that microbiological confirmation was limited to the detection of *Ureaplasma parvum* and *Mycoplasma hominis* in a single urine specimen, a non-sterile site in which both organisms may represent colonization rather than invasive infection. Molecular testing was not performed on blood, ascitic fluid, or peritoneal tissue; therefore, invasive infection could not be microbiologically confirmed. Nevertheless, the overall clinical context strongly supported presumptive targeted treatment. Supporting factors included persistent sterile pyuria, profound B-cell depletion, severe nonhepatic hyperammonemia, repeatedly negative conventional cultures, and the absence of a more compelling alternative explanation. The temporal course provided additional support: ammonia control initially remained dependent on extracorporeal clearance, with recurrent rebounds after CRRT interruptions; the brief initial exposure to levofloxacin was accompanied by a decline in plasma ammonia and C-reactive protein that reversed after treatment withdrawal; and sustained improvement followed reintroduction of levofloxacin with doxycycline, allowing CRRT to be discontinued without further rebound. This challenge-dechallenge-rechallenge pattern is consistent with an infection-related mechanism. Although antimicrobial therapy, CRRT, and surgical drainage and lavage overlapped temporally, the collective findings support the working diagnosis of a presumed invasive infection and indicate that targeted antimicrobial therapy was an important component of recovery.

A further limitation concerns the exclusion of a late-onset urea-cycle disorder. Plasma amino acids were obtained during active hyperammonemia, although after the peak of the crisis, and genetic testing was not performed. A partial urea cycle defect therefore cannot be excluded with absolute certainty. Nevertheless, the biochemical profile showed no characteristic pattern of a primary urea-cycle disorder, and the absence of previous hyperammonemic episodes, the presence of a plausible infectious trigger, and complete resolution without recurrence strongly argued against this diagnosis.

## Conclusions

Nonhepatic hyperammonemia is a rare but rapidly progressive and life-threatening complication associated with *Ureaplasma* species and *Mycoplasma hominis* infection. These organisms should be considered in immunosuppressed patients presenting with sterile pyuria, persistently negative conventional cultures, and otherwise unexplained hyperammonemia. Because they are not reliably detected by routine bacterial culture, early targeted culture or nucleic acid amplification testing may reduce diagnostic delay, particularly when the clinical presentation mimics malignancy or another inflammatory disorder.

Management requires rapid ammonia reduction, including extracorporeal clearance in severe cases, together with antimicrobial therapy active against these cell-wall-deficient organisms. In the present case, sustained metabolic recovery followed surgical drainage and peritoneal lavage, targeted treatment with levofloxacin and doxycycline, and discontinuation of CRRT without further ammonia rebound. The clinical course supports the working diagnosis of a presumed invasive infection and indicates that targeted antimicrobial therapy was an important component of recovery. Greater awareness of this syndrome among clinicians caring for patients receiving biologic immunosuppression may facilitate earlier diagnosis and improve outcomes.
